# Some Remarks on the Fever That Occurred among the Troops at Port Royal, Jamaica, during the Months of March, April, May, and June, 1819

**Published:** 1820-04

**Authors:** 

**Affiliations:** Ordnance Surgeon in that Island.


FOR THE LONDON MEDICAL AND PHYSICAL JOURNAL.

***Some Remarks on the Fever that occurred among the Troops at Port Royal, Jamaica, during the Months of March, April, May, and June,* 1819. By Dr. Miller, Ordnance Surgeon in that Island.†**

FOR several years the garrison of Port Royal has enjoyed an unusual exemption from disease, even during the period which is generally supposed to be the most unhealthy at this port, viz. from the middle of March to the end of June: and this was more especially the case during the year 1818; for, though nineteen cases of fever were admitted into the hospital, none of them had a fatal termination. This garrison had usually consisted of the head-quarters of the Royal Artillery, and now and then of a company or two of infantry. But, since the re-establishment of peace, the strength of the Royal Artillery in this island being much diminished, it has been found necessary to have always a detachment of some other regiment. Until the commencement of the present year, the 2d West-India regiment had furnished this detachment; but, as that regiment was about to leave the island, two companies of the 58th regiment were sent to Port Royal on the 27th of January. Fourteen

---

† We have received this valuable Memoir immediately from Dr. Weble, of the General Ordnance Department; and we shall take the liberty of transcribing from the letter with which it was accompanied, Dr. Weble's remarks on the character of the author.—Edit.

"The accompanying remarks on the fever which prevailed among the troops at Port Royal, Jamaica, during the months of March, April, May, and June, 1819, were sent to me immediately afterwards, by Dr. Miller, the ordnance surgeon in that island. As I was much interested by the perusal of the paper, and knew that the fullest confidence might be placed in the writer's veracity, correctness of observation, and soundness of judgment, I requested, and have obtained (however reluctantly), his consent to put it into your hands; which I did, from the persuasion that you would consider it an instructive record, and worthy of insertion in your Journal."

men of the Royal Artillery arrived from England on the 14th of February; and sixteen men of the 58th regiment, who came to the island by the same opportunity, joined their companions here on the 27th of March; and, on the 1st of May, the garrison was increased by eighty-five men of the York Chasseurs from Stoney-hill.

The barracks are situated nearly at the extremity of the long tongue of land which, running to the westward for seven or eight miles, forms the southern side of the harbour of Kingston, and then turning to the south, becomes the eastern side of that of Port Royal. This tongue is formed chiefly of a ridge of coral rock, covered with barren sand; no where more than a few feet above the level of the sea, and, where the town and barracks are situated, not more than from twelve to eighteen inches above high-water. The soldiers' barrack, intended to hold four hundred men, forms three sides of an irregular square, the side towards the south-east being open. The upper floor is good, surrounded by a balcony, and well ventilated. The lower floor is raised about two feet from the ground, but the flooring is not good: there is no passage for air underneath it, nor are there means of removing whatever may have made its way under it. The room itself is low, nor can it at all times be sufficiently ventilated.

Fever began to appear among the detachment of the 58th regiment about the vernal equinox, attacking chiefly men who had been debilitated by former disease, or had suffered from the injurious effects of ardent spirits. In the first cases, the inflammatory diathesis was not remarkable, and the lancet did not seem to produce much benefit, nor could the abstraction of blood be carried to any length. Gastric irritation was the prominent symptom, and in the fatal cases there was distinct and well-marked black vomit. About the 3d or 4th of April, the disease appeared at a stand; but recommenced with increased violence about the 12th or 14th. The subjects it now selected were the lately-arrived men of the Artillery and 58th regiment. In these cases there were high marks of inflammatory action: flushed face, blood-shot eyes, violent pains of the forehead, &c. Great relief was almost instantly produced by blood-letting. An attempt at remission seemed frequently to take place on the second or third morning; but this was too often followed by symptoms of diseased stomach, which no remedies could overpower. Of these lately-arrived men, fourteen were attacked with fever, and not fewer than eleven perished. About the 9th of May, the remainder who had as yet escaped were moved to other ports. There seemed now some cessation of disease; but, about the 13th, it made its appearance among the York Chasseurs, who had, on the 1st of that month, come to

Port Royal from Stoney-hill, thus experiencing a very considerable change of climate. Some cases in this corps put on that train of symptoms which is supposed to mark venous congestion; but, in the greater number, the active inflammatory symptoms prevailed. About the 6th or 7th of June, the virulence of the disease seems to have yielded; for, since that period, no very severe cases have occurred.

The symptoms of this disease distinctly marked it to be the epidemic remittent, or yellow-fever of the West Indies. The attack was generally sudden, the patient having perceived no premonitory symptom, except now and then some degree of costiveness. It sometimes commenced with slight rigors; but almost intolerable head-ach and pain of the back were the most urgent symptoms. The head-ach was chiefly confined to the temples and parts above the orbits. The eyes had a watery, suffused, and blood-shot appearance. Even if the patient had been almost immediately seen, the tongue had put on a whitish colour, which generally changed to a brownish-yellow, or sometimes seemed as if covered with a layer of greyish potter's clay. The countenance, at first, had an anxious and slightly-collapsed appearance, but soon became much flushed. The skin was hot and pungent, especially about the chest. The pulse was full and bounding. There was generally some degree of nausea, and sometimes vomiting of bilious matter.

These symptoms were generally much alleviated by the means used, and sometimes on the third day a period was put to the disease. In most cases there was a show of remission at this time; after which, when the fever was again aggravated, a continuance of antiphlogistic regimen sometimes restored health; and, when mercury was used, if it now began to affect the salivary glands, a favourable termination might be expected. But frequently, with great restlessness, there came on eructations, nausea, vomiting, and uneasiness on pressing the abdomen. The skin became of a dirty-yellow colour. The tongue was black, and sometimes appeared as if it had been seared with a red-hot iron. Blood oozed from the gums, and the countenance seemed much depressed. To these, as a precursor of death, the black vomit generally succeeded;—that fatal symptom which has been supposed diagnostic of a peculiar disease.

Such were the general features of this disease. Of course, the symptoms varied considerably in different individuals. In one case the patient was cut off, apparently by the effects of the disease on the brain. In some few cases black vomit did not take place; but, on examination, the stomach was found diseased, and the morbid secretion had evidently commenced. In one case from the York Chasseurs, the blood itself (of which a very small quantity only could be taken) seemed to be greatly changed. On standing some time in the cup into which it had been received, a brownish-yellow film formed on the top, but very different in appearance from the inflammatory buffy coat; the serum lay under this and the red globules at the bottom, but without any vestige of fibrin. In this case there appeared to be congestion in the lungs. On the second day the patient was seized suddenly with oppressed breathing, and very soon after expired.

On examining the bodies after death by dissection, the head generally shewed marks of increased determination of blood, with slight adhesions, and effusions of water into the ventricles. To this there was a remarkable exception, in a case where, as already mentioned, the chief morbid action seemed to be in the head; but, on removing the skull-cap, no morbid appearances could be discovered, nor did the vessels seem too turgid with blood. The lungs were now and then found to have small spots in a state of inflammation. The stomach and intestines always displayed marks of inflammation; and, in one case, I observed two or three vessels ruptured, and the orifices covered with clots of blood. But the traces of inflammation were by no means so great as one would have expected from the previous violence of the symptoms; not improbably from the cause assigned by Dr. Physic, (as quoted by Dr. Bancroft,) that "the secretion of black vomit appears to be one of the most common modes in which violent inflammation of the stomach has a disposition to terminate. Death however, in general, takes place before it has entirely disappeared." A nearly similar cause has been given by Dr. Armstrong, (*on Typhus*, p. 48;) for, not finding in the dead body great marks of inflammation, which previous symptoms had led him to expect; "it is perhaps," he says, "not improbable that this viscus [the liver], and other important parts, occasionally lose their vitality from inflammation, and yet, on examination, exhibit no signs of the previous excitement; all signs of that having passed away before death by an excess of *morbid secretion*." The liver was in general not diseased; and the gall-bladder and its bile did not appear to differ from the natural and healthy state. The urinary bladder was generally empty. Indeed, it appears that this secretion, and almost all the natural ones, seem to stop after the commencement of the black vomit; as if nature had thrown its whole secreting energy into the diseased stomach. It is extremely probable, from the violent pains of the back, that in some cases the medulla spinalis was diseased; especially in the case of an artillery-man, during the last day of whose life the lower extremities were painful to an extraordinary degree, and before death became almost paralytic. I regretted much that I had not means to lay open that canal.

In treating this disease, my first endeavour was to reduce the very great inflammatory action which almost universally was present. For this purpose, the lancet (of the safety, nay, absolute necessity, of which, I have had abundant proofs) was used to the utmost extent that the pulse admitted, and that no inconsiderable experience in the use of this remedy would permit. Its use was not regulated by the quantity of blood taken, but by the effects on the patient, who generally experienced great relief from this evacuation; and in several instances the disease was cut short by it. But it was too often impossible to remove the inflammatory tendency in the stomach, even when the bleeding was pushed to the utmost extent that the patient's strength allowed. In some cases where, on the patient being taken into the hospital, and after the free use of the lancet, there was any bilious vomiting, the stomach was cleansed out with warm water, and, if this did not appear sufficient, by giving half a drachm of the sulphate of zinc, which, from its immediate operation, I preferred. I am by no means friendly to the general use of emetics in this disease, and think that I have often seen them hurtful when composed of antimony or ipecacuanha; but the method I used on this occasion was frequently useful, and never, as far as I can judge, produced any deleterious effects. The bowels were carefully evacuated by submuriate of mercury, combined either with jalap or compound extract of colocynth; and they were kept constantly free, by repeating the same medicines, or by solutions of neutral salts. The cold affusion and sponging were constantly employed when the heat of the skin was great. These applications were highly grateful to the patients, and also very useful; though perhaps not so much so as if water of a cooler temperature could have been procured, it being seldom found at less than 80°. Blisters to the head, the neck, the spine, and the stomach, were constantly in use; as also cataplasms of mustard and pepper over the latter organ. In such cases as appeared from the first to be dangerous, endeavours were made to saturate the system with mercury. To this remedy I was formerly by no means partial; but the opinions of Dr. Armstrong and others, with their arguments, had removed some prejudices which I felt against it. It was also a favourite of, and strongly recommended by, the late Dr. M'Kenzie. Some advantage was unquestionably derived from its use, especially in cases where there was torpor, and as if an effusion were within the head. Where the salivary glands became affected, the patient might in general be pronounced safe: but one exception to this took place, in a gunner of the Royal Artillery, in whom a flow of saliva, after twelve hours' continuance, receded; gastric irritation supervened, and death followed. In some cases, the gums became spongy, and large quantities of blood were discharged, without mixture of saliva. This was a bad, though not always a fatal, symptom. I am, however, in considerable doubt whether mercury, given in the large quantity which is required, did not on some occasions act as a poison; and, when taken by the mouth in the form of a submuriate, whether it did not sometimes increase the gastric inflammation. To these may be added the use of the warm bath, of purgative glysters, of stimulating frictions, of wine, &c. as circumstances seemed to require. Bark was frequently tried, when there were no contraindications to its use; but I cannot say that, on this occasion, I found the same benefit from it as at other times when the remissions were more distinct.

Such were the principal means used for combating this disease; and, though the success has not been such as to boast of, I am not aware that any better plan could be adopted. "It has not been my lot," as Dr. Johnson says, "to find intertropical fevers so very tractable as some medical officers have, or say they have, found them."

It is now necessary to enquire from what cause this disease appeared, at a period of the year *(saluberrimum ver)* which is generally supposed, at least in warm climates, to be the most healthy. To this, however, Port Royal has always been an exception; for, when sickness has prevailed here, it has usually been between the months of May and June. Some observations relative to this fact are to be found in Staff-surgeon Doughty's work on Yellow Fever. I am not aware that any attempts have been made to explain the causes of this exception. Whether the causes which appear to me to have operated during this season, have also been those which produced similar effects at other times, I am not sufficiently informed to determine.

To close one question entirely, I must state, that neither now, nor at any former period, have I ever perceived any circumstances connected with the epidemic fever of the West Indies, to induce me to look upon it as contagious.

We must, then, have recourse to the other great cause of epidemic fever, for the explanation we are seeking, viz. the miasmata produced by the decomposition of vegetable matter, or of vegetable and animal matter combined: what has been called, rather indiscriminately, marsh miasmata. On stating this as the cause of fever at Port Royal, a query naturally arises, how, in such a place, so situated on a barren sand, and without any marsh in its vicinity, can there be grounds for attributing fever to such a cause? To this we may reply, that, though the ground on which Port Royal stands does not of itself furnish much vegetable matter, this may be, and is, brought to it, in like manner as to a ship, on-board of which it has been completely proved that fever has originated from the decomposition of matters brought on-board. I presume that it will be readily granted, that, after several months' continuance of dry weather, there must be considerable quantities of vegetable and animal matters scattered about in the neighbourhood of a barrack, containing a numerous body of men, women, and children, and which it may be totally impossible for our senses to perceive. I am inclined to think, from causes already mentioned, that some degree of this occurs under the lower floor of the barracks at Port Royal.

From whence then does it happen, that this accumulation of vegetable and animal substances, which must take place every dry season, one year produces violent disease, and during another is apparently harmless? It seems highly probable, that, when the weather during the spring is perfectly regular, with constant alternations of sea-breeze and land-wind, by which the heat of the day is counterbalanced by the coolness of the night, then no decomposition takes place, and no noxious miasmata are evolved; especially if there be no cause inducing moisture. It is very probable (if my information be correct) that, during this season, Fort Augusta, a situation not very dissimilar to Port Royal, has escaped disease from having frequently had a land-wind and cool nights; whilst at Port Royal it was perfectly calm, and the nights were almost intolerably hot. But, completely to answer this question, it would perhaps be necessary to compare the temperature, &c. of many different years; but for which I have no documents. Without however going far back, it will not be uninteresting, to compare together the state of the weather during the months of March, April, May, and to the 20th of June, of the years 1818 and 1819; during the former of which periods, nineteen men were admitted with fever into the Royal-Ordnance Hospital, out of which none died; whilst, during the latter, one hundred and twenty cases of fever occurred, and twenty-seven fell victims to its ravages. It is unfortunate that, in order to keep the journal of which the following statement is an abstract, I am not in possession of a register-thermometer, so as to have been able to note the lowest degree at which the instrument stood during the twenty-four hours. It is not difficult to note the highest, as it generally occurs at an hour or two after noon; but, to have observed the lowest during the night, could not have been done, without much trouble and uncertainty. Had this been done, I am convinced that the average lowest degree would have been in 1818 much below that in 1819. The universal remark of the great heat of the nights, is a proof of this, though perhaps not an accurate one. But it will be seen that, for a much greater proportion of the nights in 1819, the sea-breeze continued to blow all night; and it is well known that this is much warmer than the land-wind, so that when it sets in, even after an hour or two of calm, with a burning sun, forming to the sensations by much the most unpleasant time of the day, the thermometer is raised two or three degrees.

MARCH 1818.

| | |
|---|---|
| 1 day thermometer stood at | 81° |
| 7 days ................ | 83 |
| 7 — ................ | 84 |
| 13 — ................ | 85 |
| 2 — ................ | 86 |
| 1 — ................ | 87 |

Average greatest height, $84\frac{1}{3}$°.
7 days strong gales,
14 nights land-wind,
14 nights calm,
3 nights sea-breeze,
1 day slight rain.

MARCH 1819.

| | |
|---|---|
| 2 days thermometer stood at | 83° |
| 6 — ................ | 84 |
| 18 — ................ | 85 |
| 3 — ................ | 86 |
| 2 — ................ | 87 |

Average greatest height, 85°.
21 days strong gales,
6 nights land-wind,
20 nights calm,
5 nights sea-breeze,
2 days slight rain.

APRIL 1818.

| | |
|---|---|
| 3 days thermometer stood at | 84° |
| 11 — ................ | 85 |
| 10 — ................ | 86 |
| 6 — ................ | 87 |

Average greatest height, $85\frac{1}{3}$°.
2 days strong gales,
7 nights land-wind,
23 nights calm,
5 days rain.

APRIL 1819.

| | |
|---|---|
| 8 days thermometer stood at | 83° |
| 3 — ................ | 84 |
| 4 — ................ | 85 |
| 12 — ................ | 86 |
| 1 — ................ | 88 |
| 2 — ................ | 89 |

Average greatest height, $85\frac{1}{7}$.
26 days strong gales,
2 nights land-wind,
28 nights calm,
3 days rain.

MAY 1818.

| | |
|---|---|
| 1 day thermometer stood at | 78° |
| 4 days ................ | 86 |
| 19 — ................ | 87 |
| 6 — ................ | 88 |
| 1 — ................ | 90 |

Average greatest height, 87°.
17 days strong gales,
14 nights land-wind,
16 nights calm,
1 night sea-bseeze,
7 days rain.

MAY 1819.

| | |
|---|---|
| 6 days thermometer stood at | 86° |
| 6 — ................ | 87 |
| 13 — ................ | 88 |
| 6 — ................ | 89 |

Average greatest height, 87°$\frac{3}{5}$.
14 days strong gales,
4 nights land-wind,
19 nights calm,
8 nights sea-breeze,
6 days rain.

JUNE 1818.

| Days | | Thermometer |
|---|---|---|
| 7 days | thermometer stood at | 86° |
| 3 — | ................ | 87 |
| 9 — | ................ | 98 |
| 1 — | ................ | 0½ |

Average greatest height, 87¼°.
6 days strong gales,
5 nights land-wind,
11 nights calm,
4 nights sea-breeze,
5 days rain.

JUNE 1819.

| Days | | Thermometer |
|---|---|---|
| 1 day | thermometer stood at | 80° |
| 1 — | ................ | 84 |
| 2 days | ................ | 85 |
| 6 — | ................ | 86 |
| 4 — | ................ | 87 |
| 6 — | ................ | 88 |

Average greatest height, 86⅓.
13 days strong gales,
1 night land-wind,
9 nights calm,
10 nights sea-breeze,
4 days rain.

From these tables it appears, that the average greatest heat, except during the month of June, was somewhat higher during 1819 than in 1818; and, for the reason formerly stated, as well as from the comparatively few nights on which there was a land-wind, I cannot doubt but that the average lowest temperature was much less in 1818 than in 1819. The number of days on which strong gales blew, was this year much greater than during the last, and in violence they much exceeded what had happened for at least the preceding five years; even so as to render the passage to Kingston, if not dangerous, at least very disagreeable, which had rarely been the case formerly during this period of the year. Violent breezes appear frequently to have been the precursors or companions of epidemic fever; but it seems to me probable, that, through their means, we derived the moisture which was necessary to produce the decomposition of the vegetable matters. Strong breezes from the southward and eastward have the effect of causing an inflow of water into the harbour, by which the tide is raised considerably, and the water, percolating through the sand, is brought nearer to the surface. That this is the case, we have often ocular demonstration, from the moist appearance of the parade, without any previous fall of rain; and I observed, about the end of last March, that the water rose in a hole which had been dug, to within six or seven inches of the surface. Thus, from the frequent and strong south-east gales during this season, there must have been a constant supply of water to the surface; and in proof of this it may be stated, that, on having some of the flooring of the lower barrack raised, traces of moisture were there very evident. Besides, this effect was kept up by the rare occurrence of the land-wind, by which the nights are not only cooled, but a counteraction is produced on the water in the harbour.

Perhaps it may be said, as another objection to the opinion I have offered, that, as these remains of vegetable and animal matter must be constantly found at Port Royal after dry weather, how happens it that, after rains, sickness does not always occur? To this it may be replied, that, in general, when there has been no sickness previous to the spring seasons (as the rains are here called), some happens after, as was the case in 1817. But it appears to me probable, that a considerable quantity of the materials which would become noxious, is, from the heavy falls of rain, swept into the sea.

There is another circumstance which might assist, not in producing disease, but in rendering the human body more susceptible to its influence. It is well known to all who have resided for any considerable time at Port Royal, that, when a sea-breeze blows all night, the sleep, if any, is much disturbed, the skin feels parched, and one rises in the morning feeling as if the sleep we may have had, had not brought with it its usual refreshing powers.

It therefore appears to me that we owe the intensity of disease during this spring to the general increase of temperature, and to the uncommonly boisterous weather, acting in the manner I have stated.

I am not aware that any means could be adopted, which in future might render us more independant of the state of the weather, unless perhaps that it might be useful to have the lower floor raised, the parts under thoroughly cleaned, and the boarding repaired. It might also be useful to open air-holes at each end from without, so as to cause some degree of ventilation underneath.

*Port Royal; June* 28, 1819.

